# Insect-Flower Interaction Network Structure Is Resilient to a Temporary Pulse of Floral Resources from Invasive *Rhododendron ponticum*

**DOI:** 10.1371/journal.pone.0119733

**Published:** 2015-03-12

**Authors:** Erin Jo Tiedeken, Jane C. Stout

**Affiliations:** 1 Botany Department, School of Natural Sciences, Trinity College Dublin, Dublin 2, Ireland; 2 Trinity Centre for Biodiversity Research, Trinity College Dublin, Dublin 2, Ireland; Central China Normal University, CHINA

## Abstract

Invasive alien plants can compete with native plants for resources, and may ultimately decrease native plant diversity and/or abundance in invaded sites. This could have consequences for native mutualistic interactions, such as pollination. Although invasive plants often become highly connected in plant-pollinator interaction networks, in temperate climates they usually only flower for part of the season. Unless sufficient alternative plants flower outside this period, whole-season floral resources may be reduced by invasion. We hypothesized that the cessation of flowering of a dominant invasive plant would lead to dramatic, seasonal compositional changes in plant-pollinator communities, and subsequent changes in network structure. We investigated variation in floral resources, flower-visiting insect communities, and interaction networks during and after the flowering of invasive *Rhododendron ponticum* in four invaded Irish woodland sites. Floral resources decreased significantly after *R*. *ponticum* flowering, but the magnitude of the decrease varied among sites. Neither insect abundance nor richness varied between the two periods (during and after *R*. *ponticum* flowering), yet insect community composition was distinct, mostly due to a significant reduction in *Bombus* abundance after flowering. During flowering *R*. *ponticum* was frequently visited by *Bombus*; after flowering, these highly mobile pollinators presumably left to find alternative floral resources. Despite compositional changes, however, network structural properties remained stable after *R*. *ponticum* flowering ceased: generality increased, but quantitative connectance, interaction evenness, vulnerability, H’_2_ and network size did not change. This is likely because after *R*. *ponticum* flowering, two to three alternative plant species became prominent in networks and insects increased their diet breadth, as indicated by the increase in network-level generality. We conclude that network structure is robust to seasonal changes in floral abundance at sites invaded by alien, mass-flowering plant species, as long as alternative floral resources remain throughout the season to support the flower-visiting community.

## Introduction

In light of the variety of threats facing plant and pollinator populations [1], understanding and preserving plant-pollinator interactions has become increasingly important. Recently, some studies have moved beyond a single-species approach and instead utilize the analysis of ecological networks to better understand the structure of entire plant-pollinator communities [2–5]. This work has identified some common properties of plant-pollinator network structure; for example, networks often display nestedness (they have a core group of generalists that interact with one another, with specialists mostly interacting with a subset of species interacting with generalist species), and asymmetry (specialist plants interact with generalist pollinators, and vice versa) [6,7]. It is also widely accepted that plant-pollinator interactions are largely generalized; the existence of extreme specialists is rarer than once thought [8,9]. These properties are thought to increase the stability and robustness of networks [10,11], especially when faced with species extinctions [12,13]. Studies of network structure are also used to examine perturbations to networks [14], such as invasion by alien plants and pollinators.

Quantitative network studies have demonstrated that invasive plants tend to be integrated into native plant-pollinator networks through native or invasive generalist flower-visitors that incorporate the alien into their diets [2,15,16]. Many invasive plants have flowers which are functionally simple, with large nectar rewards [17,18]. They thus often form strong connections with a large proportion of pollinating species and can receive more visits than co-flowering plants [19–21], potentially altering network properties such as the distribution of interactions among species in the community (interaction evenness) [22]. Nevertheless, networks often appear to retain characteristics of robust communities even after invasion by alien plants [22–24].

An inherent limitation to community-level studies investigating impacts of invasive alien plants on native plant-pollinator networks is locating comparable, uninvaded control sites, and ensuring that floral abundance is not a confounding factor between invaded and uninvaded sites [20,25]. Current studies have dealt with these limitations by surveying areas that exhibit only initial stages of invasion [20,24], by comparing invaded and flower removal plots [21], and by investigating sites along an invasion gradient [22,25]. However most studies survey plant-pollinator communities either exclusively during the flowering period of the invasive species [20,21,23], or summarize network structure over the entire flowering-season [2,15,19]. Very few account for within-season temporal dynamics in network structure [but see 22,26].

With showy floral displays and copious sugar-rich nectar production, invasive alien plants that occur at high relative abundances could be functionally similar to mass-flowering crop species: they may provide a floral pulse that could be a valuable resource to generalist pollinators [27–30]. For example, the mass-flowering crop oil seed rape has been shown to increase the densities and colony growth of bumblebee species [29,30]. In temporal climates in particular, however, the floral resources provided by many invasive plants are temporary because they tend to flower for a relatively short portion of the overall flowering season. No study to date has considered seasonal variation in floral resources in communities invaded by an alien plant species. If floral resource availability decreases enough after the cessation of flowering of an invasive species, obligate flower-visiting insects that relied heavily on the invasive plant could be negatively affected, resulting in changes to network structure. Alternatively, if remaining floral resources are sufficient to sustain the pollinator community, network structure may remain relatively unchanged. The same patterns could result after the flowering of a highly abundant native plant species, however invasive plants have been shown to decrease native plant abundance and diversity [31,32], which decreases the chances of a consistent, reliable flower supply throughout the season. In this study, we investigated how floral resources and insect-flower interaction community structure change after the flowering period of an abundant invasive plant species.

*Rhododendron ponticum* is a severely invasive alien plant species in north-western Europe. It was introduced to the United Kingdom and subsequently Ireland in the eighteenth century as an ornamental species and as game cover [33]. An evergreen, perennial shrub, *R*. *ponticum* invades Irish heaths, bogs, and particularly woodlands, where it can alter native plant community composition [34]. *R*. *ponticum* presents large floral displays comprised of inflorescences with 9–21 pink-purple zygomorphic flowers [35]. These flowers produce a large amount of sugar-rich nectar, making them very attractive to native flower-visitors [36,37]. Studies on the reproductive biology of *R*. *ponticum* in its invasive range demonstrate that the plant is visited by a variety of insect taxa but pollinated mainly by generalist bumblebee (*Bombus*) species [38]. Recent work has shown that invasive *R*. *ponticum* contains high concentrations of a class of plant secondary compounds, usually associated with defense against herbivory, in its floral nectar [39]. These secondary compounds (diterpenes known as grayanotoxins) are toxic to some pollinating insect species in the plant’s invasive range, including honeybees and some solitary bees (personal observation). Because *R*. *ponticum* nectar is toxic to some flower-visitors, when in flower, this invasive plant may provide a significant floral resource pulse to only part of the flower-visiting community.

Using a quantitative analytical approach, this study aimed to investigate the role of *R*. *ponticum* in four invaded woodland communities in southeast Ireland, and to determine how the insect community responds to changes in floral resource abundance and composition. We surveyed floral abundance and conducted focal observations of the entire flowering plant community while *R*. *ponticum* was in flower and again after flowering of the invasive ceased, in order to investigate changes in insect-flower communities during these two distinct time periods. Specifically we aimed to test the following hypotheses: (1) that floral resource availability decreased at invaded sites after the cessation of *R*. *ponticum* flowering, (2) that obligate flower-visiting insect diversity, abundance, and visitation rates were higher during vs. after *R*. *ponticum* flowering, and that insect community composition differed during the two periods, and (3) that insect-flower interaction network structure (i.e. size, connectance, evenness, weighted plant and animal linkage, and level of specialization) changed after the cessation of *R*. *ponticum* flowering, with smaller and more fragmented networks after *R*. *ponticum* flowering.

## Materials and Methods

### Study sites

Observations of insect-flower interactions were carried out at four native mixed or oak woodland forest sites invaded by *R*. *ponticum* (Co. Wicklow, southeast Ireland, Table 1). In order to standardize abiotic conditions and plant communities, sites were selected that were similar in aspect, elevation, and invasion intensity of *R*. *ponticum* (*R*. *ponticum* plant cover accounted for approximately one third of the total area of each site, 33.2% ± 8.2% (mean ± SD); coverage estimates were obtained using 20 x 20 m quadrats). Sites were on average 22.33 ± 9.83 km apart to reduce the possible overlap of pollinator communities based on their predicted foraging ranges [40]. Because *R*. *ponticum* requires high light intensity in order to germinate, it often invades forests where there has been a disturbance that causes openings in the canopy (i.e. tree felling), or at edge habitats created by streams or roads [34]. At our study sites additional flowering species often occurred near these edges as well as in clearings in the forest. Sites were thus defined as 100 x 50 m areas incorporating the portion of the forest invaded by *R*. *ponticum* as well as any edge habitat that bordered the invaded area.

**Table 1:** Study site characteristics.

Site	Location	Altitude (m)	Sampling round	Dominant alternative plant species
Crossover	52.894 N 6.400 E	165	R1	*Hyacinthoides non-scripta, Ulex europaeus*
			R2	*Rubus fruticosus, Galium aparine*
Dunran	53.060 N 6.102 E	160	R1	*Cytisus scoparius ssp scoparius, Veronica chamaedrys*
			R2	*Stachys sylvatica, Digitalis purpurea*
Shankhill	53.192 N 6.427 E	284	R1	*Stellaria holostea, V. chamaedrys*
			R2	*D. purpurea, G. aparine*
Trooperstown	53.017 N 6.274 E	185	R1	*H. non-scripta, V. chamaedrys*
			R2	*S. sylvatica, D. purpurea*

Characteristics of four study sites in southeast Ireland, County Wicklow, including dominant alternative (non-*Rhododendron*) plant species during (R1) and after (R2) flowering of invasive *Rhododendron ponticum*.

### Plant and insect sampling

In 2011, each site was sampled on at least three distinct days during *R*. *ponticum* flowering (24 May–28 June, hereafter referred to as **sampling round one**) and again immediately after *R*. *ponticum* flowering ended (4–26 July, hereafter referred to as **sampling round two**). Communities were sampled using the timed observation method [15,22,41]. Timed observations help alleviate the bias of overestimating the degree of specialization of rare plants by standardizing observation times [42]. Observations of each plant species were made on at least three distinct days during each sampling period and each day of sampling comprised 3 × 10 min observations, (morning (9:00–12:00), midday (12:00–14:30), and afternoon (14:30–17:30)) in order to account for any temporal variation in visitation patterns. Thus we aimed to observe each plant species for a total of 1.5 hours/site/sampling period. Inclement weather and differences in flowering phenology reduced the total observation time/species to an average of 1.23 ±0.44 h, however relatively limited sampling effort has been shown to capture a large proportion of the functionally most important community members in plant-pollinator networks [43]. Observations were carried out on dry days when the temperature was > 12°C and wind speeds were ≤ 4 according to the Beaufort Scale.

During our censuses, we recorded the identity of all diurnal, obligate flower-visitors to flowering branches (shrubs and treelets) or flower patches (herbs) [44]. Although facultative flower-visitors (including beetles and some Dipteran species) may play a role in pollination and plant-pollinator network structure, obligate visitors, including bees (Hymenoptera: Apidae), hoverflies (Diptera: Syrphidae), and butterflies (Lepidoptera), are often the most important and effective pollinators of wild and crop plants [45–47]. In addition, because they rely completely on floral resources for food as adults, they are most likely to be affected by changes in floral abundance and were thus the focus of this study. The number of floral units visited by each individual visitor (visitation) as well as the number of individuals of each species (abundance) was recorded. A visit (synonymous with interaction) was defined as any contact between the flower and the insect. The number of floral units observed during each census and the total number of floral units visited by each insect was noted. A floral unit was defined as a single flower head, or part of a multiple head, from which a medium-sized bee has to fly rather than walk to reach another floral unit of the same species [48].

Where possible, insects were identified on the wing in the field. Unknown specimens were captured and identified to the lowest possible taxonomic category (usually species level). *Bombus lucorum*, *Bombus cryptarum*, *Bombus magnus* and *Bombus terrestris* are part of the *Bombus sensu stricto* species complex, and were thus grouped as “*B*. *lucorum aggregate*” because of their morphological similarity [49,50]; a previous study found that approximately two-thirds of individuals of this aggregate in this area are *B*. *lucorum* [51]. Bumblebees, hoverflies and butterflies were identified to species level using the appropriate field guides and keys [52,53], except for certain hoverfly genera that were difficult to distinguish. *Melanostoma/Platycheirus* species were grouped together because of their morphological similarity, and species of *Xylota*, *Syrphus*, and *Meliscaeva* were identified to genus only. Subsequent sampling at the site however demonstrated that the number of species from each of these genera were low, and thus unlikely to affect network structure. An insect reference collection is deposited at Trinity College Dublin. Flowering plant identification followed Parnell and Curtis [54] and Rose [55].

We collected floral abundance data at our sites in order to investigate changes in floral resources between the two rounds of sampling, and to weight interactions by the abundance of flowering plant species [22,56]. Established *R*. *ponticum* grows in dense stands that make random quadrat sampling at sites impossible. Instead, our sampling method was a stratified randomized approach, reflecting the relative abundance of *R*. *ponticum* at each site (approximately one third cover). Eight 10 m transects were established in areas free from *R*. *ponticum* cover, and the number of floral units of each “non-*Rhododendron*” species was recorded in three 1 x 1m quadrats along each transect (at 0, 5 and 10 m, 24 quadrats). To sample floral abundance in the area covered by *R*. *ponticum*, twelve 1 x 1 m quadrats were placed at waist height on twelve randomly selected *R*. *ponticum* plants and the number of floral units in each counted. This sampling method was replicated three times throughout each period of sampling at each site at the same time as insect observations were made. The floral abundance of each species was calculated by dividing the total number of flowers by the total number of quadrats sampled at each site (mean number of flowers/m^2^), and was used in order to weight networks by the relative abundance of each flowering plant species [22].

We used the total number of observations of each insect species as a measure of abundance of insects at our sites [20,22]. In addition to total insect abundance and richness, a number of other parameters were also compared between sampling rounds including a.) bumblebee abundance, b.) bumblebee richness, c.) hoverfly abundance, and d.) hoverfly richness. Solitary bees and butterflies were too rare at sites to be analyzed, but were included in interaction networks (see next section).

### Insect-flower interaction networks

We constructed two fully quantitative interaction matrices for each site, one for each round of sampling. Following the methodology of Kaiser-Bunbury et al. [22], we used mean interaction frequencies in our data matrices to account for slight differences in sampling effort between plant species at a site. We used interaction frequencies to represent interaction strength between plant and insect species, and quantified visits based on the floral abundance of the interaction partner; ‘mean interaction frequency’ was represented as the total number of visits /flower/hour of animal species *a* to plant species *p* multiplied by the floral abundance (average floral units/ m^2^) of plant species *p* [11,22,57,58]. Due to the small size of our daily networks, data from each of the three visits to a site were combined and networks and network parameters were calculated at the site level for each sampling period [25]. Mean interaction frequencies of flower-visitors at each site were also used to construct non-metric multi-dimensional scaling (nMDS) plots in order to investigate patterns of flower-visiting insect communities.

For comparison with other networks, we calculated qualitative network parameters for our sites during each sampling round (Table 2) following Dorman et al. [7]. We also calculated quantitative network descriptors in order to compare the structure of the insect-flower interaction networks between the two sampling periods. Quantitative as opposed to qualitative parameters incorporate the interaction frequency of individual species and are preferable because they are more robust than their qualitative equivalents to variations in sampling effort and changes in network size [59,60]. Using the “networklevel” command in the bipartite package [61] in R (version 3.0.2, R-Development-Core-Team, 2007[62]), we calculated: 1. **Quantitative connectance** (the realized proportion of all possible links weighted by the quantitative visitation rate of each species, [22,59], calculated as **linkage density**/species richness (P+A). Connectance is a measure of species richness and has been shown to increase the rate and stability of ecosystem processes such as pollination [14]); 2. **Interaction evenness** (a measure of how well distributed interactions are among species within communities, based on the Shannon index and calculated as IE = p_pa_log2p_pa_/log2S, where S = total number of insect-flower interactions in the network and p_pa_ is the proportion of interactions between plant *p* and animal *a* [22,60]. Interaction evenness can describe patterns of interaction strengths in the network, which are important because networks with many weak interactions are thought to be more stable [14]); 3. **Generality** (or the weighted linkage for insect visitors, used to represent the level of generalization in the diets of pollinators [13] and calculated as the weighted mean number of plant species per visitor species); 4. **Vulnerability** (or the weighted linkage for plants, calculated as the weighted mean number of insect visitor taxa per plant species [22]); and 5. **H’**_**2**_ (a measure of the overall level of specialization in a network, ranges between 0 (no specialization) and 1 (perfect specialization), calculated based on the difference between realized and expected interactions [7]. More generalized networks have higher redundancy and are therefore thought to withstand species extinctions better than specilized networks [14]).

**Table 2:** Qualitative network parameters.

		Number of plant taxa (*P*)	Number of insect taxa (*A*)	Number of links (*L*)	Number of visits (*V*)	Ratio (*A/P*)	Network size (*S*)	Connectance (*C*)^1^	Maximal plant linkage (*l*_*max*_)	Maximal animal linkage (l_max_)	Mean plant linkage (l_p_)	Mean animal linkage (l_a_)
Crossover	R1	10	16	53	180	1.60	160	313.13	11*	9	5.30 ± 3.40	3.31 ± 2.68
	R2	11	17	66	299	1.55	187	35.29	11	10	6.00± 2.82	3.88 ± 3.25
Dunran	R1	13	15	54	179	1.15	195	27.69	12*	10	4.15 ± 2.73	3.60 ± 2.67
	R2	11	15	45	195	1.36	165	27.27	9	10	4.09 ± 3.01	3.00 ± 2.62
Shankhill	R1	10	19	52	200	1.90	190	27.37	14*	10	5.20 ± 3.61	2.74 ± 2.70
	R2	12	16	59	178	1.33	192	30.73	13	10	4.92 ± 3.34	3.69 ± 2.85
Trooperstown	R1	9	13	30	116	1.44	117	25.64	8*	8	3.33 ± 2.12	2.31 ± 2.25
	R2	9	16	43	99	1.78	144	29.86	10	8	4.78 ± 3.23	2.69 ± 2.06

Qualitative network parameters for insect-flower interaction networks during (R1) and after (R2) flowering of invasive *Rhododendron ponticum*. Qualitative network parameters include the number of plant species (*P*), number of flower-visiting insect species (*A*), the total number of unique flower-insect interactions (links, *L*), the total number of interactions between plants and insects (interactions, *I*), ratio of animal to plant species (*A*/*P*), full network size (*S* = A*P), qualitative connectance (*C* = 100* *L*/*S*) and mean and maximum plant and animal linkage.

* indicates that *R*. *ponticum* was the plant species with the highest linkage in the network.

### Data analysis

Network parameters and insect and taxon-specific abundance, visitation, and richness, were calculated as mean values per sampling round, averaged across all four sites; thus, they were compared between the two sampling rounds using univariate analyses (paired t-tests). The limited power associated with our low sample size (n = 4 networks per sampling round) is largely justified by the considerable effort involved in sampling entire insect-flower interaction communities in a limited time period (during the flowering period of the invasive species), and is not unusual for similar studies [20,21].

Floral abundance data were analyzed using mixed effects models in SPSS (response variable = floral units per meter^2^). Sampling round (during or after flowering), site (1–4), and their interaction were included in the model as fixed factors, and quadrat nested within site was included as a random factor. In order to investigate how *R*. *ponticum* influences floral resources available to obligate flower-visitors, two separate models were run; one for the total floral units recorded (complete model), and one for only non-*Rhododendron* floral units. Models were validated by plotting standardised residuals against fitted values, and floral abundance was log +1 transformed where necessary. Fisher LSD post hoc comparisons were used to compare floral abundance at each site during the two sampling rounds.

Differences in the composition of available floral resources between the two rounds of sampling were visualised using non-metric multi-dimensional scaling (nMDS) plots based on Bray-Curtis dissimilarity matrices in PRIMER 6 (Version 6.1.13) (Plymouth Routines in Multivariate Ecological Research, Plymouth Marine Laboratory, Plymouth, UK). Floral unit data were square root transformed in order to prevent highly abundant plant species (i.e *R*. *ponticum*) from dominating the analyses. We tested for differences in floral composition between the two sampling rounds using non-parametric multivariate analysis of variance (Permutational MANOVA, “PERMANOVA”), with sampling round included as a fixed factor and site as a random factor. The PRIMER routine SIMPER (Similarity of Percentages) analysis was used to identify which species were important in distinguishing among communities from the different rounds of sampling. SIMPER tables (see S3 and S4) included species until a cumulative 70% of dissimilarity was accounted for. The same multivariate techniques and model design used for the floral abundance data were employed in order to investigate differences in patterns of mean insect visitation frequencies.

### Ethics Statement

Study sites were located in forests owned by the state-sponsored private company Coillte (locations: 52.894 N-6.400 W, 53.060 N-6.102 W, 53.192 N-6.427 W, and 53.017 N-6.274 W, coordinate system WGS84), and all necessary permissions were obtained prior to the study. For future permissions contact Coillte headquarters at 00-353-12011111, or visit their website (http://www.coillte.ie/). No permits were required for insect sampling but we complied with good research practices throughout the study. Our field studies did not involve any endangered or protected species. Data on insect and plant surveys are deposited in Ireland’s National Biodiversity Data Centre.

## Results

In total, 1,446 insect-flower interactions were observed in approximately 108 hours of focal observations during the two rounds of sampling. Of those visits, 675 and 771 were observed during and after *R*. *ponticum* flowering respectively. Floral visitors were comprised of insects from three Orders; Diptera (the most species-rich group, 16 syrphid taxa), Hymenoptera (five bumblebee species, one honeybee species, and two solitary bee genera), and Lepidoptera (one butterfly and one moth species) (S1 Table). The syrphids accounted for the majority of overall interactions at our sites (78.8%), followed by the bees (20.2%) and butterflies and moths (1.6%). All observed insect taxa were native to Ireland.

### 1- Changes in floral abundance

**Fig 1:**
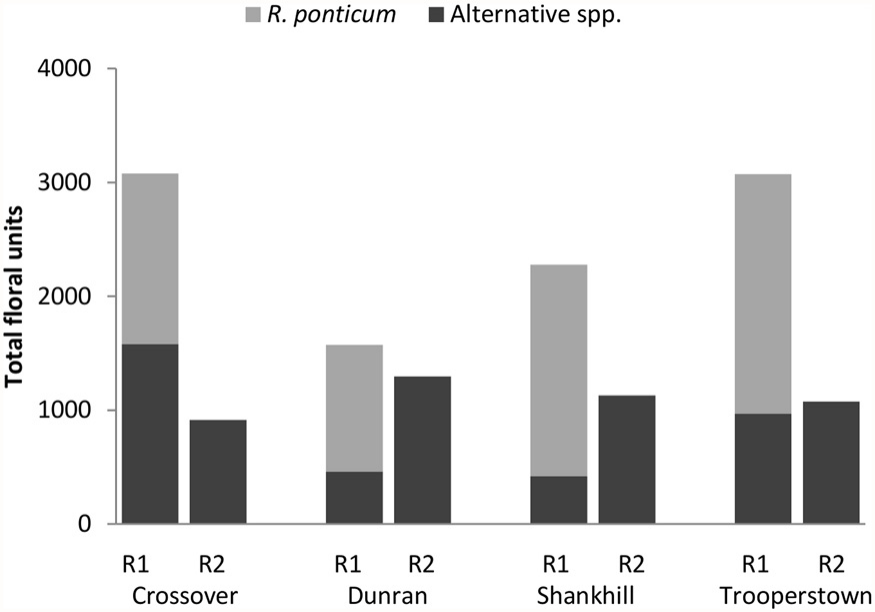
Floral abundance, during and after *R*. *ponticum* flowering Total number of available floral units, comprised of invasive *R*. *ponticum* (light grey bars) and alternative plant species (dark grey bars), during the flowering of *R*. *ponticum* (R1) and after cessation of the flowering of the invasive species (R2) at four invaded Irish woodland sites.

A total of 35 plant species were observed during the two sampling rounds, 25 species during *R*. *ponticum* flowering and 20 species after flowering ceased, with 10 species flowering during both rounds (S2 Table). Plant species richness at sites did not vary significantly between the two sampling rounds (paired t-test: t = 0.3, d.f. = 3, p = 0.790). During *R*. *ponticum* flowering, *Hyacinthoides non-scripta* and *Cytisus scoparius ssp*. *scoparius* were often the most abundant non-*Rhododendron* flowers, although their mean floral abundance per m^2^ was 2–10 times less than that of *R*. *ponticum*’s. In the second round of sampling, the average floral abundance per m^2^ of all flowering plant species was low in comparison to *R*. *ponticum* in the first round, but *Rubus fruticosus*, *Stachys sylvatica*, and *Digitalis purpurea* flowers were often the most abundant species (Table 1). While *R*. *ponticum* was in flower, (sampling round 1) it comprised on average just over two-thirds of the total available floral units (average 67.37% ± 13.7, Fig. 1).

Overall, mean floral units per m^2^ decreased significantly after *R*. *ponticum* stopped flowering (F_1, 716_ = 30.363, p < 0.01, Fig. 1). The magnitude of this decrease, however, depended on the site being sampled (site*sampling round interaction, F_3, 716_ = 2.939, p < 0.05). Post hoc comparisons using the Fisher LSD test revealed that there was a significant decrease in floral units per m^2^ at all sites (Crossover: p < 0.01, Shankhill: p < 0.05, Trooperstown: p < 0.01) except for Dunran (p = 0.586). There was no consistent pattern in abundance of non-*Rhododendron* flowering units among sites between rounds (site*sampling round interaction, F_3, 476_ = 4.095, p < 0.01): Fisher LSD post hoc comparisons revealed a decrease in non-*Rhododendron* floral units at Crossover (p = 0.050), an increase at Dunran (p = <0.05) and Shankhill (p < 0.05), and no significant change at Trooperstown (p = 0.755) (Fig. 1).

**Fig 2:**
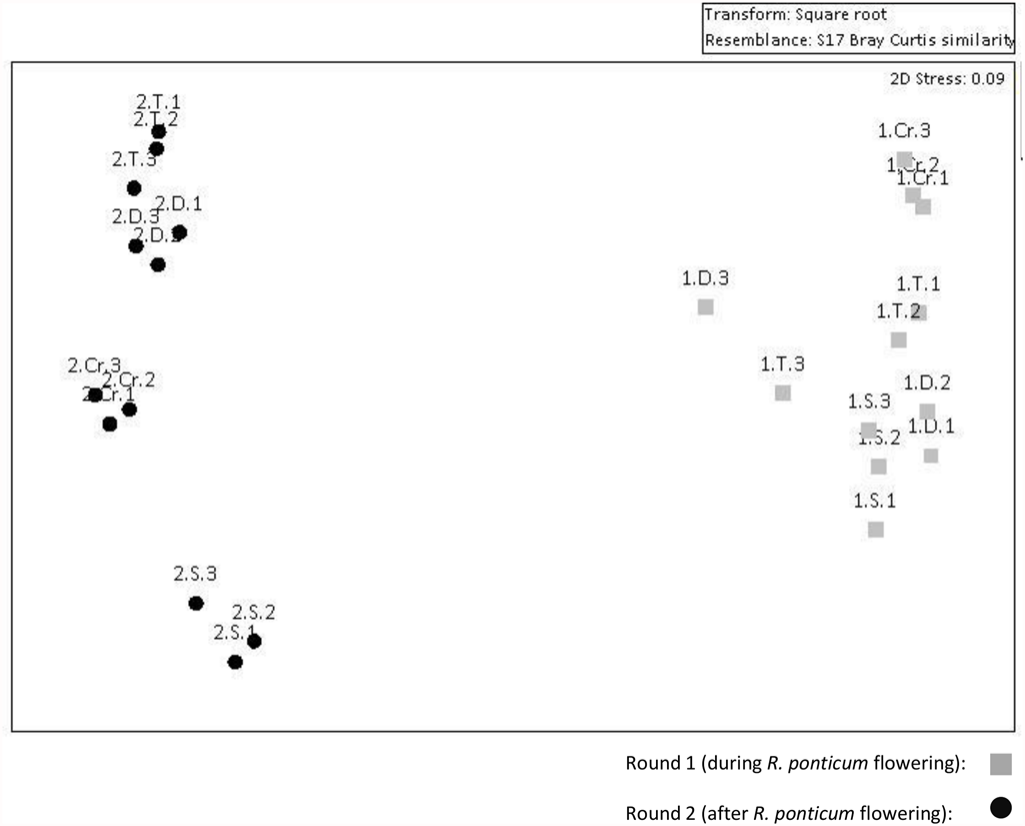
Non-metric Multi-Dimensional Scaling plot of floral abundance data Each point on the graph represents a replicate of floral abundance sampling, n = 3 replicates per site per sampling round. The closer the points, the more similar the identity and abundance of flowering plant species. Light grey squares represent sites sampled during *R*. *ponticum* flowering and black circles represent the same sites after flowering of the invasive species ceased. Label codes indicate the sampling round (1 or 2), the site name (C = Crossover, D = Dunran, S = Shankhill, T = Trooperstown), and the replicate (1, 2, or 3). Data were square root transformed to balance contributions of rarer and dominant flowering species.

Multivariate analysis showed the composition of the floral resources available to obligate flower-visitors during round one (when *R*. *ponticum* was in flower) was significantly different from that of round two (after flowering ceased, main effect sampling round: F_1, 3_ = 10.58, p < 0.05). This difference is of course mostly attributed to the cessation of *R*. *ponticum* flowering, but also to the start of flowering of a few abundant alternative plant species, namely *Stachys sylvatica*, *Digitalis purpurea* and *Rubus fruticosus* (Fig. 2, S3 Table). The model also revealed a significant main effect of site (F_3, 16_ = 19.29, p < 0.01) and a significant site*sampling round interaction (F_3, 16_ = 9.70, p < 0.01); flowering communities were more distinct between sites after *R*. *ponticum* flowering, and more similar during flowering.

### 2- Changes in flower-visitor diversity, visitation, and composition

**Fig 3:**
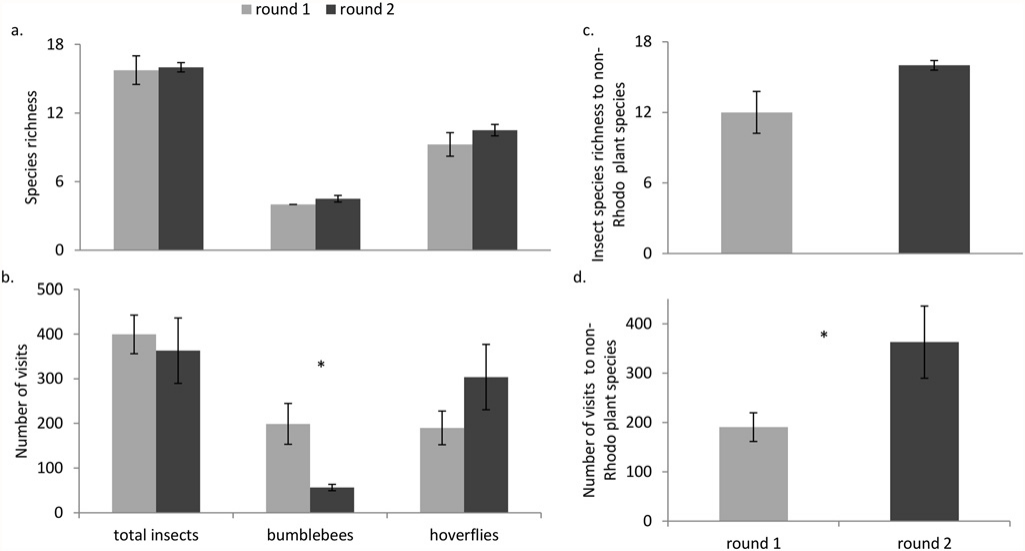
Flower-visitor species richness and abundance data Flower-visitor species richness and abundance during *R*. *ponticum* flowering (round 1, light grey bars) and after the cessation of flowering of the invasive (round 2, dark grey bars). Comparison between round 1 and 2 of a.) total insect, bumblebee, and hoverfly species richness and b.) number of visits of total insects, bumblebees, and hoverflies, c.) insect species richness to non-*Rhododendron* plants, and d.) number of visits to non-*Rhododendron* plants. Plots show mean values per round, averaged across the sites, and vertical bars show the standard error of each mean. Number of visits represents the number of floral units visited by insects, however insect abundance (number of individuals, not shown) followed the same patterns. Significant differences in the above parameters when comparing the two rounds of sampling are indicated by * (paired t-test, p < 0.05).

Overall, total insect visits (TIV) and insect species richness (ISR) at sites did not differ between the two sampling rounds (TIV: t = 0.777, ISR t = 0.200, d.f. = 3, p > 0.05, Fig. 3a & b). The total visits observed to non-*Rhododendron* plant species however, increased significantly after the cessation of *R*. *ponticum* flowering (t = 3.674, d.f = 3, p < 0. 05, Fig. 3d). Insect species richness to non-*Rhododendron* flowering plants was not significantly different between the two sampling rounds (t = 2.376, d.f. = 3, p = 0. 098, Fig. 3c). The number of visits observed from bumblebees decreased at our sites after the cessation of *R*. *ponticum* flowering (t = 3.449, p < 0.05); however, bumblebee and hoverfly species richness and observed hoverfly visits did not change significantly (bumblebee richness: t = 1.732, hoverfly richness: t = 1.058, hoverfly visits: t = 1.657, d.f. = 3, p = > 0.05, Fig. 3a & b).

**Fig 4:**
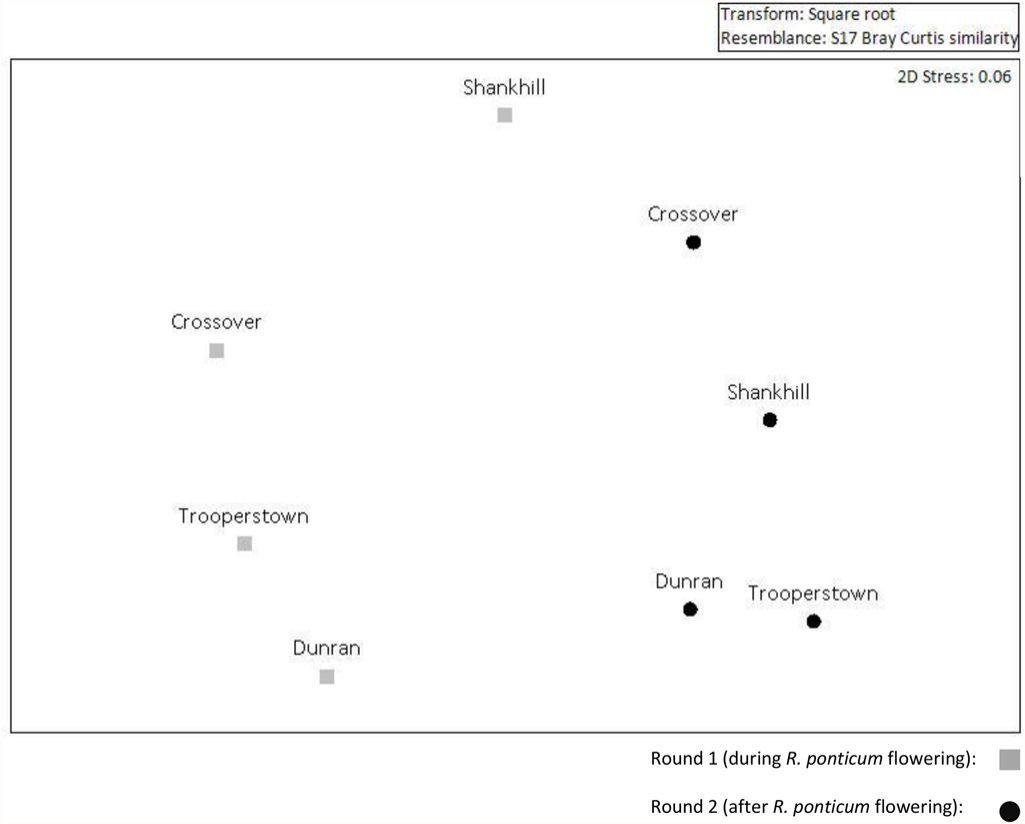
Non-metric Multi-Dimensional Scaling plot of flower-visitor interaction frequencies Flower-visitor composition at sites invaded by *R*. *ponticum* during (round 1) and after (round 2) flowering. Site level mean interaction frequencies of pollinator groups were used to calculate the resemblance matrix. The closer the points, the more similar the identity and abundance of interaction frequencies of flower-visitors recorded. Data were square root transformed to balance contributions of rarer and dominant insect visitors.

Multivariate analysis revealed that the mean interaction frequencies of insect communities observed during sampling round one (when *R*. *ponticum* was in flower) were significantly different from those observed in sampling round two (F_1, 3_ = 4.538, p < 0.05, Fig. 4). The main contributors to this difference were the bumblebees and hoverflies in the genera *Meliscaeva* and *Sphegina*; the mean interaction frequency of *B*. *lucorum agg*. and *Sphegina clunipes* decreased once *R*. *ponticum* flowering ceased, while that of *Meliscaeva* increased (S4 Table).

### 3- Changes in insect-flower interaction networks

Networks from both sampling rounds were small (minimum of nine plant species and 13 animal species, maximum of 13 plant species and 19 animal species, Table 2), but network size did not differ significantly between the two sampling rounds (paired t-test, t = 0.480, d.f = 3, p = 0.664).

**Fig 5:**
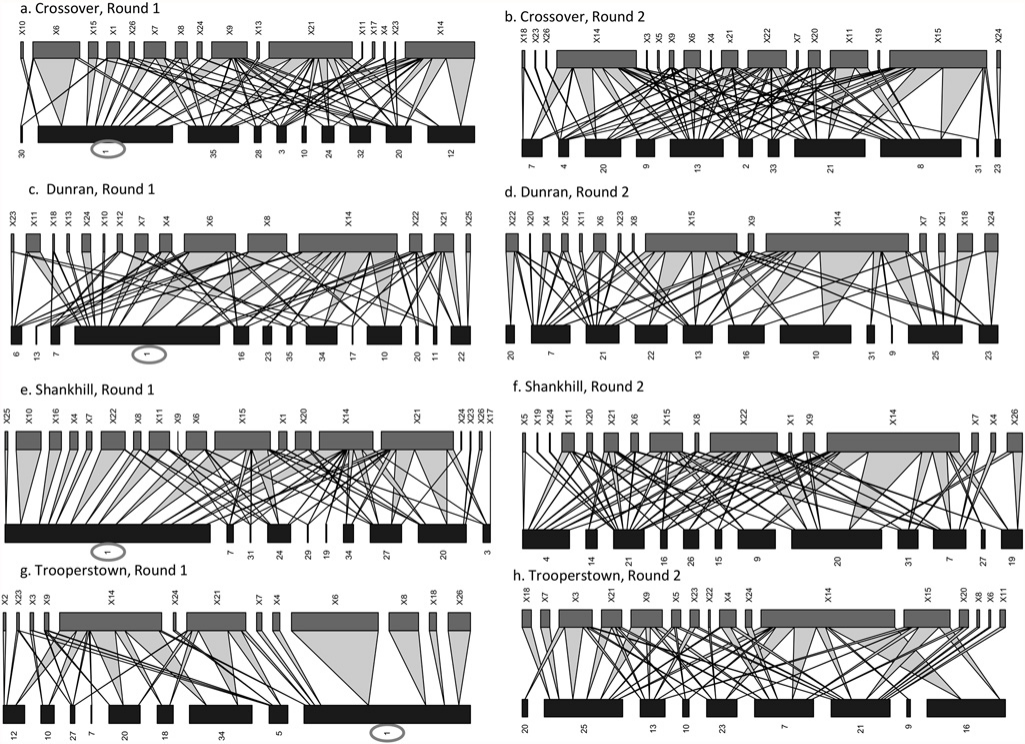
Quantitative insect-flower interaction networks Networks represent insect-flower communities at four Irish woodland sites during (a, c, e, g) and after (b, d, f, h) *R*. *ponticum* flowering. For each web, upper bar widths represent pollinator guild abundance while lower bar widths are determined by the interaction strength with insect species. On the lower bars, the number 1 corresponds to *R*. *ponticum* and is circled in gray; remaining species codes and pollinator guild abbreviations are listed in S1 & S2 Tables. Linkage width indicates the frequency of the interaction. One network was calculated for each site during each round of sampling.

During its flowering period, *R*. *ponticum* was highly connected and dominant in insect-flower interaction networks at our sites (Fig. 5a, c, e & g). It was the plant with the highest linkage in all four sites (Table 2), interacting with on average 74.10% (± 13.1) of flower visiting species. Bumblebees were the most common visitors to *R*. *ponticum*, but visits from hoverflies were also common. R. *ponticum* also dominated the networks in terms of visitation: 55.65% ± 8.04 of all interactions were to *R*. *ponticum*. None of the round one networks exhibited significant compartmentalization (Fig. 5a, c, e & g), and the majority (average 75.23% of species ±16.6) of insect species interacting with *R*. *ponticum* also interacted with at least one additional plant species.

**Fig 6:**
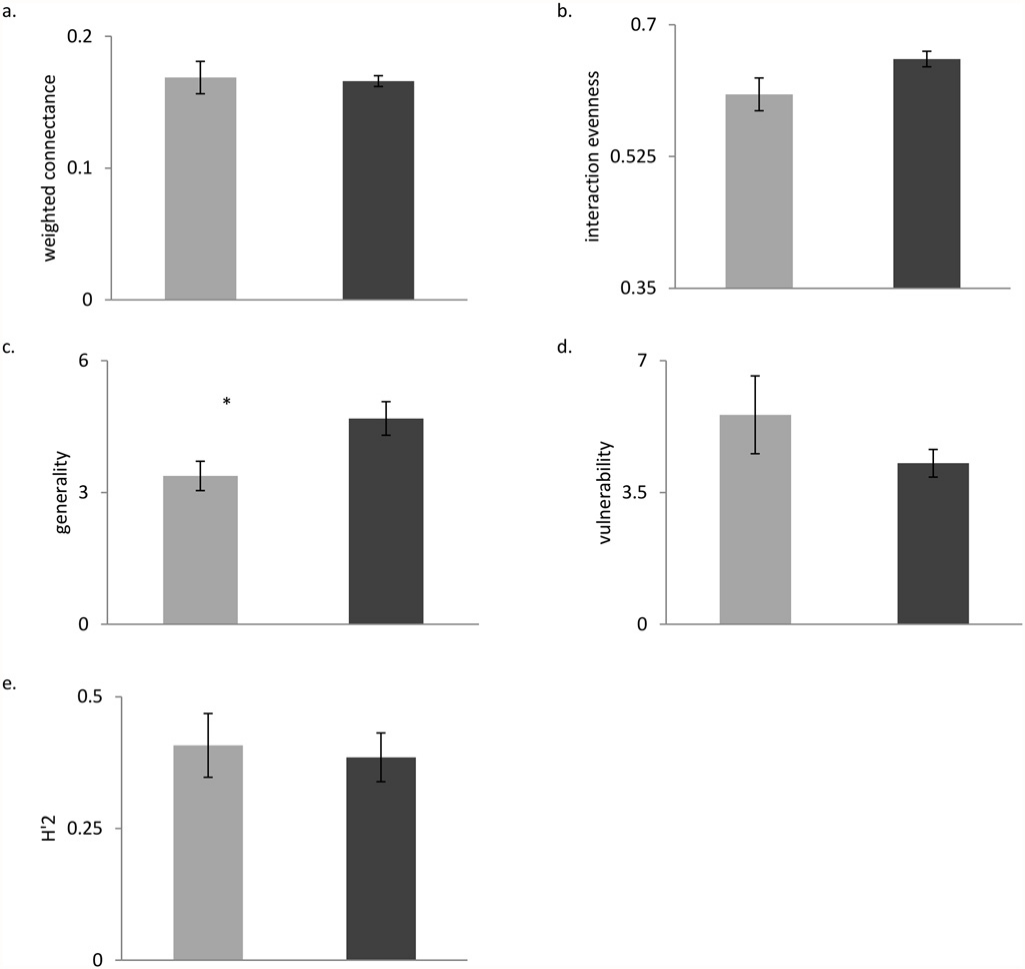
Quantitative network parameters Comparison of quantitative network parameters calculated from insect-flower interaction networks created by sampling four invaded Irish woodland insect- communities during (light gray) and after (dark gray) flowering of *Rhododendron ponticum*. Network parameters analyzed include a.) quantitative connectance, b.) interaction evenness, c.) generality, d.) vulnerability and e.) H’_2_. * indicates a significant difference between the two rounds of sampling (paired t-test, p < 0.05). Plots show mean parameters per sampling round across all four sites, and vertical bars show the standard error of each mean.

Of the quantitative network parameters calculated for each site, only generality differed between the two sampling rounds, increasing significantly after *R*. *ponticum* stopped flowering (t = -3.516, d.f. = 3, p < 0.05, Fig. 6c, Fig. 5). In contrast, quantitative connectance, interaction evenness, vulnerability, and H’_2_ did not change significantly after *R*. *ponticum* stopped flowering (QC: t = 0.123, IE: t = -2.251, V: t = 1.060, H’_2_: t = 0.457, d.f. = 3, p >0.05; Fig. 6a–b, d–e).

## Discussion

When in flower, *R*. *ponticum* is highly connected and dominant in native insect-flower interaction networks. Our study, however, demonstrates that despite changes in the composition of communities after *R*. *ponticum* stops flowering, insect-flower interaction network structure at moderately invaded sites remains robust,.

### 1- Changes in floral abundance

As predicted, sites experienced a significant decrease in overall floral abundance after *R*. *ponticum* stopped flowering. After *R*. *ponticum* flowering, the abundance and diversity of plant species that remain or come into flower next dictate the severity of the impact of this decline in total floral abundance. Invasive plants often compete with native plants and change plant species composition, resulting in a decrease in plant diversity and abundance [32,34,63]. Thus, the abundance of flowering species in invaded locations could be low. Surprisingly, our study demonstrates that this is not always the case. There can be significant variation in alternative floral resource abundance among sites, even when the level of invasion is consistent. Dunran and Shankhill both had an increase in alternative (non-*Rhododendron*) floral units after *R*. *ponticum* flowering ceased, Trooperstown experienced no significant change, and Crossover saw an overall decrease in alternative floral units. Crossover, however, had a much higher number of alternative floral units during *R*. *ponticum* flowering in comparison to the other sites; thus, although the decrease in round 2 was significant, the overall floral availability was still comparable to the other sites. While our sites were representative of invaded woodlands on the east coast of Ireland, it should be noted that *R*. *ponticum* cover in woodlands in the west and other habitat types (bogs, heathland) can be substantially higher [25,36,64]. A more consistent and severe decrease in non-*Rhododendron* floral resources may be expected at these heavily invaded sites. Our study is the first to measure seasonal fluctuation in floral resources at sites invaded by an alien, mass-flowering plant species, and to consider how these fluctuations may directly impact obligate flower-visitors.

### 2- Changes in flower-visitor diversity, visitation, and composition

Total insect abundance and richness at our sites did not change significantly between the two sampling periods, however the number of visits to co-flowering, non-*Rhododendron* species increased significantly when *R*. *ponticum* was no longer in flower. The majority of studies investigating the impact of invasive alien plants on native co-flowering plant pollination find primarily negative effects [65,66]. Our findings suggest that negative impacts on the pollination of co-flowering plants may not persist throughout the flowering season, however, further studies investigating pollen deposition and seed set are required to test this hypothesis. Furthermore, relative changes in floral abundance at the sites during the two sampling rounds could have an impact on visitation rates. At sites where total floral abundance decreased significantly (Crossover, Shankhill and Trooperstown), increased visitation rates might be expected, since total insect abundance did not change significantly. The smaller relative change in total floral abundance between sampling rounds at Dunran, however, could impact visitation rates to native flowers at this site.

Even though total insect abundance and species richness did not change between the two sampling rounds, the composition of the insect communities visiting flowers was distinct, largely due to a decrease in bumblebee visitation after the cessation of *R*. *ponticum* flowering. In our networks, the links between bumblebees and *R*. *ponticum* were strong during sampling round one. Bumblebee richness remained similarly low (5 species) in the second sampling period, however the abundance and visitation of bumblebees at the sites dropped drastically, indicating that *R*. *ponticum* is an important forage resource for bumblebees [37,67]. Some mass flowering agricultural crops have previously been shown to increase the density and colony growth of bumblebee species [28,29]; abundant invasive *R*. *ponticum* may provide a similarly important resource for this genera. The change in the composition of the insect communities after *R*. *ponticum* stopped flowering may simply have been due to seasonal variation in the abundance or activity of different insect species. However long-season, generalist bumblebees could have left the sites after *R*. *ponticum* stopped flowering to find more rewarding or abundant forage sources elsewhere [68]. Bumblebees are efficient foragers, have large foraging ranges [40], and are able to utilize resources distributed across a landscape scale [69]. Other insects, such as hoverflies, may not be capable of such long-range foraging.

### 3- Changes in insect-flower interaction networks

During its flowering period, *R*. *ponticum* was highly connected in insect-flower interaction networks and dominated network structure. It received, on average, half of the overall insect visits at sites, and was by far the most highly connected plant species. This finding is consistent with previous investigations of communities invaded by alien plant species; for example, three other invasive plants with large showy floral displays, *Impatiens glandulifera*, *Carpobrotus affine acinaciformis*, and *Opuntia stricta*, were also integrated into network structure, to the point where they received significantly more pollinator visits or higher visitation rates than co-flowering native species [20,26]. To our knowledge however, none of these invasive plants expressed traits that made their rewards unavailable to a large proportion of members of the pollinator community. Even the concealed nectar of *I*. *glandulifera* is exploited by a wide range of insects [21]. *R*. *ponticum* nectar, in contrast, is toxic to honeybees and at least one solitary bee species (genus *Andrena*) in its invasive range (personal observation). Generalist honeybees are often frequent visitors of invasive plant species, and can significantly alter network structure [22]. The absence of honeybees from *R*. *ponticum* invaded networks, presumably due to the toxic effects of *R*. *ponticum* nectar, could therefore impact species interactions and levels of connectance between community members. Regardless of its toxic nectar, however, *R*. *ponticum* still acted as a supergeneralist species in our networks [19,20].

Despite the decrease in floral resources and the compositional changes to the community, our results demonstrate that network structure remained stable after *R*. *ponticum* finished flowering. Only flower-visitor generality, or the weighted mean number of plant species per insect species, changed significantly between sampling rounds; it increased after *R*. *ponticum* stopped flowering. This is probably because no single alternative species replaced *R*. *ponticum* in terms of dominance of the network. Instead two to three plant species became more prominent in networks. Flower-visitors therefore included more plant species in their diets after the flowering of the invasive species, presumably to obtain sufficient floral resources.

Studies have shown decreases in network size, and visitor species richness and abundance when invasive flowers are removed from invaded sites [21], and differences in interaction evenness (the distribution of interactions between different species in the network) among sites varying in invasion intensity [22]. Furthermore, models which have simulated species removal in order to investigate the impact of species loss on network structure have demonstrated that loss of highly connected community members leads to network collapse quicker than loss of less connected species [13,70]. We therefore hypothesized that the structure of invaded networks would change once abundant, highly connected *R*. *ponticum* stopped flowering. On the contrary, network structure remained relatively stable, probably because there was the opportunity for the insect and floral communities to respond to compositional changes (re-wiring) [12,71] which was not the case in a previous study [13]. Similar to our findings, several recent studies of temporal variation in uninvaded plant-pollinator communities have shown that although the composition of communities changes within and between seasons, network structural properties remain relatively consistent due to re-wiring [41,72–74]. The temporal variation exhibited by our networks was therefore similar to that of uninvaded networks, regardless of the floral resource pulse provided by *R*. *ponticum*. Our work supports previous findings that networks are resilient to invasion by alien plant species [22–24], even after considering the significant seasonal variation in floral resources in invaded communities. This may not be the case at more heavily invaded sites [25], where native plant diversity could be severely depleted and therefore unable to sustain the insect community after the flowering of the invasive species.

Our results may also be useful from a conservation perspective. Invasive alien plant species are often cleared in order to benefit biological diversity and allow the recovery of ecosystems [75]. If invasive plants integrate into networks and strongly interact with flower-visiting species, their removal could have important and potential detrimental effects on the pollinator community that relied on the invasive as a floral resource, particularly if native flowering plants are not restored [76]. Our results indicate that at least for moderately invaded sites, if *R*. *ponticum* was removed for conservation purposes, network structure may be resilient to the loss of this highly connected invasive plant.

## Conclusions

Our findings demonstrate that an entomophilous invasive alien plant can integrate into native insect-flower interaction networks, even when the floral rewards it provides are not suitable for the entire flower-visiting community. Our work also demonstrates that although the composition of flowering plant and insect communities changes at sites after an abundant invasive plant species stops flowering, community structure can remain relatively stable if the flower-visitor community expands its diet and utilizes available alternative floral resources. We conclude that the seasonal impacts of invasion by alien plants on insect-flower interaction networks are dependent not only on the traits of the invasive species but the composition of the native plant community.

## Supporting Information

S1 Table Pollinator species. Species codes and long hand for pollinator guilds represented in Fig. 5.
(DOCX)

S2 Table Plant species. Species codes and long hand for plants represented in Fig. 5.
(DOCX)

S3 Table Plant species SIMPER analysis. The contribution of each plant species to the composition of floral resources at sites invaded by *R*. *ponticum* in round 1 vs. 2 of sampling as determined by SIMPER (Similarity of Percentages) analysis. Data were square root transformed.
(DOCX)

S4 Table Pollinator species SIMPER analysis. The contribution of each insect taxon to communities at sites invaded by *R*. *ponticum* in round 1 vs. 2 of sampling, as determined by SIMPER (Similarity of Percentages) analysis. Data were square root transformed.
(DOCX)

## References

[1] Kearns CA, Inouye DW, Waser NM. Endangered mutualisms: the conservation of plant-pollinator interactions. Annu Rev Ecol Syst. 1998;29: 83–112.

[2] Memmott J, Waser NM. Integration of alien plants into a native flower—pollinator visitation web. Proc R Soc Lond B Biol Sci. 2002;269: 2395–2399. 10.1098/rspb.2002.2174 PMC1691186 12495480

[3] Vázquez DP, Blüthgen N, Cagnolo L, Chacoff NP. Uniting pattern and process in plant—animal mutualistic networks: a review. Ann Bot. 2009;103: 1445–1457. 10.1093/aob/mcp057 19304996 PMC2701748

[4] Jordano P. Patterns of mutualistic interactions in pollination and seed dispersal: connectance, dependence asymmetries, and coevolution. Amer Nat. 1987;657–677.

[5] Memmott J. The structure of a plant-pollinator food web. Ecol Lett. 1999;2: 276–280. 10.1046/j.1461-0248.1999.00087.x 33810635

[6] Bascompte J, Jordano P. Plant-animal mutualistic networks: the architecture of biodiversity. Ann Rev Ecol Evol Syst. 2007;38: 567–593.

[7] Dormann CF, Fründ J, Blüthgen N, Gruber B. Indices, graphs and null models: analyzing bipartite ecological networks. The Open Ecology Journal. 2009;2: 7–24.

[8] Bosch J, Martín González AM, Rodrigo A, Navarro D. Plant—pollinator networks: adding the pollinator’s perspective. Ecol Lett. 2009;12: 409–419. 10.1111/j.1461-0248.2009.01296.x 19379135

[9] Waser NM, Chittka L, Price MV, Williams NM, Ollerton J. Generalization in pollination systems, and why it matters. Ecology. 1996;77: 1043–1060.

[10] Bascompte J, Jordano P, Melián CJ, Olesen JM. The nested assembly of plant—animal mutualistic networks. Proc Natl Acad Sci USA. 2003;100: 9383–9387. 12881488 10.1073/pnas.1633576100 PMC170927

[11] Bascompte J, Jordano P, Olesen JM. Asymmetric coevolutionary networks facilitate biodiversity maintenance. Science. 2006;312: 431–433. 16627742 10.1126/science.1123412

[12] Kaiser‐Bunbury CN, Muff S, Memmott J, Müller CB, Caflisch A. The robustness of pollination networks to the loss of species and interactions: a quantitative approach incorporating pollinator behaviour. Ecol Lett. 2010;13: 442–452. 10.1111/j.1461-0248.2009.01437.x 20100244

[13] Memmott J, Wasser NM, Price MV. Tolerance of pollinaiton networks to species extinctions. Proc R Soc Lond B Biol Sci. 2004;271: 2605–2611. 10.1098/rspb.2004.2909 PMC1691904 15615687

[14] Tylianakis JM, Laliberté E, Nielsen A, Bascompte J. Conservation of species interaction networks. Biol Cons. 2010;143: 202–205.

[15] Morales CL, Aizen MA. Invasive mutualisms and the structure of plant—pollinator interactions in the temperate forests of north‐west Patagonia, Argentina. J Ecol. 2006;94: 171–180.

[16] Olesen JM, Eskildsen LI, Venkatasamy S. Invasion of pollination networks on oceanic islands: importance of invader complexes and endemic super generalists. Diversity and Distributions. 2002;8: 181–192.

[17] Ghazoul J. Flowers at the front line of invasion? Ecol Entomol. 2002;27: 638–640.

[18] Stout JC, Morales CL. Ecological impacts of invasive alien species on bees. Apidologie. 2009;40: 388–409.

[19] Aizen MA, Morales CL, Morales JM. Invasive mutualists erode native pollination webs. PLoS Biol. 2008;6: e31 10.1371/journal.pbio.0060031 18271628 PMC2235906

[20] Bartomeus I, Vila M, Santamaria L. Contrasting effects of invasive plants in plant-pollinator networks. Oecologia. 2008;155: 761–770. 10.1007/s00442-007-0946-1 18188603

[21] Lopezaraiza—Mikel ME, Hayes RB, Whalley MR, Memmott J. The impact of an alien plant on a native plant—pollinator network: an experimental approach. Ecol Lett. 2007;10: 539–550. 17542933 10.1111/j.1461-0248.2007.01055.x

[22] Kaiser‐Bunbury CN, Valentin T, Mougal J, Matatiken D, Ghazoul J. The tolerance of island plant—pollinator networks to alien plants. J Ecol. 2011;99: 202–213.

[23] Padrón B, Traveset A, Biedenweg T, Díaz D, Nogales M, et al Impact of alien plant invaders on pollination networks in two archipelagos. PLoS One. 2009;4: e6275 10.1371/journal.pone.0006275 19609437 PMC2707600

[24] Vilà M, Bartomeus I, Dietzsch AC, Petanidou T, Steffan-Dewenter I, et al Invasive plant integration into native plant-pollinator networks across Europe. Proc R Soc Lond B-Biol Sci. 2009;276: 3887–3893. 10.1098/rspb.2009.1076 PMC2817287 19692403

[25] Stout JC, Casey LM. Relative abundance of an invasive alien plant affects insect—flower interaction networks in Ireland. Acta Oecol. 2014;55: 78–85.

[26] Bartomeus I, Vila M, Steffan‐Dewenter I. Combined effects of Impatiens glandulifera invasion and landscape structure on native plant pollination. J Ecol. 2010;98: 440–450.

[27] Diekötter T, Kadoya T, Peter F, Wolters V, Jauker F. Oilseed rape crops distort plant—pollinator interactions. J Appl Ecol. 2010;47: 209–214.

[28] Stanley DA, Knight ME, Stout JC. Ecological variation in response to mass-flowering oilseed rape and surrounding landscape composition by members of a cryptic bumblebee complex. PLoS One. 2013;8: e65516 23840338 10.1371/journal.pone.0065516 PMC3686753

[29] Westphal C, Steffan‐Dewenter I, Tscharntke T. Mass flowering crops enhance pollinator densities at a landscape scale. Ecol Lett. 2003;6: 961–965.

[30] Westphal C, Steffan‐Dewenter I, Tscharntke T. Mass flowering oilseed rape improves early colony growth but not sexual reproduction of bumblebees. J Appl Ecol. 2009;46: 187–193.

[31] Levine JM, Vila M, Antonio CMD, Dukes JS, Grigulis K, et al Mechanisms underlying the impacts of exotic plant invasions. Proc R Soc Lond B-Biol Sci. 2003;270: 775–781. 10.1098/rspb.2003.2327 PMC1691311 12737654

[32] Martin PH. Norway maple (Acer platanoides) invasion of a natural forest stand: understory consequence and regeneration pattern. Biol Invasions. 1999;1: 215–222.

[33] Cross J. Biological flora of the British Isles: Rhododendron ponticum L. J Ecol. 1975;63: 345–364.

[34] Cross JR. The establishment of Rhododendron ponticum in the Killarney oakwoods, SW Ireland. J Ecol. 1981;69: 807–824.

[35] Stout JC. Reproductive biology of the invasive exotic shrub, Rhododendron ponticum *L*. (Ericaceae). Bot J Linn Soc. 2007;155: 373–381.

[36] Dietzsch AC, Stanley DA, Stout JC. Relative abundance of an invasive alien plant affects native pollination processes. Oecologia. 2011;167: 469–479. 10.1007/s00442-011-1987-z 21484398

[37] Stout JC, Parnell JAN, Arroyo J, Crowe TP. Pollination ecology and seed production of Rhododendron ponticum in native and exotic habitats. Biodivers Conserv. 2006;15: 755–777.

[38] Stout JC. Pollination of invasive Rhododendron ponticum (Ericaceae) in Ireland. Apidologie. 2007;38: 1–9.

[39] Tiedeken EJ, Stout JC, Stevenson PC, Wright GA. Bumblebees are not deterred by ecologically relevant concentrations of nectar toxins. J Exp Biol. 2014;217: 1620–1635. 10.1242/jeb.097543 24526720 PMC4006588

[40] Knight ME, Martin AP, Bishop S, Osborne JL, Hale RJ, et al An interspecific comparison of foraging range and nest density of four bumblebee (Bombus) species. Mol Ecol 2005;14: 1811–1820. 15836652 10.1111/j.1365-294X.2005.02540.x

[41] Olesen JM, Bascompte J, Elberling H, Jordano P. Temporal dynamics in a pollination network. Ecology. 2008;89: 1573–1582. 18589522 10.1890/07-0451.1

[42] Gibson RH, Knott B, Eberlein T, Memmott J. Sampling method influences the structure of plant—pollinator networks. Oikos. 2011;120: 822–831.

[43] Hegland SJ, Dunne J, Nielsen A, Memmott J. How to monitor ecological communities cost-efficiently: the example of plant—pollinator networks. Biol Conserv. 2010;143: 2092–2101.

[44] Power EF, Stout JC. Organic dairy farming: impacts on insect—flower interaction networks and pollination. J Appl Ecol. 2011;48: 561–569.

[45] Sugiura N. Pollination of the orchid Epipactis thunbergii by syrphid flies (Diptera: Syrphidae). Ecol Res. 1996;11: 249–255.

[46] Vance NC, Bernhardt P, Edens RM. Pollination and seed production in Xerophyllum tenax (Melanthiaceae) in the Cascade Range of central Oregon. Am J Bot. 2004;91: 2060–2068. 10.3732/ajb.91.12.2060 21652355

[47] Winfree R, Williams NM, Dushoff J, Kremen C. Native bees provide insurance against ongoing honey bee losses. Ecol Lett. 2007;10: 1105–1113. 17877737 10.1111/j.1461-0248.2007.01110.x

[48] Dicks LV, Corbet SA, Pywell RF. Compartmentalization in plant—insect flower visitor webs. J Anim Ecol. 2002;71: 32–43.

[49] Carolan JC, Murray TE, Fitzpatrick Ú, Crossley J, Schmidt H, et al Colour patterns do not diagnose species: quantitative evaluation of a DNA barcoded cryptic bumblebee complex. PLoS One. 2012;7: e29251 10.1371/journal.pone.0029251 22238595 PMC3253071

[50] Murray TE, Fitzpatrick U, Brown MJF, Paxton RJ. Cryptic species diversity in a widespread bumble bee complex revealed using mitochondrial DNA RFLPs. Conserv Genet. 2008;9: 653–666.

[51] Byrne E. Ecological, molecular and morphological characterization of cyptic bumblebees. 2010. MSc Thesis, Trinity College Dublin.

[52] National Biodiversity Data Centre. Identification Guide to Ireland’s Bumblebees. 2010 Waterford: National Biodiversity Data Centre http://pollinators.biodiversityireland.ie/id-guides/. Accessed January 2011.

[53] Stubbs AE, Falk SJ. British Hoverflies: An Illustrated Identification Guide. 1983 London: British Entomological and Natural History Society.

[54] Parnell J, Curtis T. Webb’s an Irish Flora. 8th edition 2011 Cork: Cork University Press.

[55] Rose F, O’Reilly C. The Wild Flower Key: How to Identify Wild Flowers, Trees and Shrubs in Britain and Ireland. 2006 London: The Penguin Group.

[56] Kaiser-Bunbury CN, Memmott J, Müller CB. Community structure of pollination webs of Mauritian heathland habitats. Perspect Plant Ecol Evol Syst. 2009;11: 241–254.

[57] Vázquez DP, Melián CJ, Williams NM, Blüthgen N, Krasnov BR, et al Species abundance and asymmetric interaction strength in ecological networks. Oikos. 2007;116: 1120–1127.

[58] Vázquez DP, Morris WF, Jordano P. Interaction frequency as a surrogate for the total effect of animal mutualists on plants. Ecol Lett. 2005;8: 1088–1094.

[59] Bersier L-F, Banašek-Richter C, Cattin M-F. Quantitative descriptors of food-web matrices. Ecology. 2002;83: 2394–2407.

[60] Tylianakis JM, Tscharntke T, Lewis OT. Habitat modification alters the structure of tropical host-parasitoid food webs. Nature. 2007;445: 202–205. 17215842 10.1038/nature05429

[61] Dormann CF, Gruber B, Fründ J. Introducing the bipartite package: analysing ecological networks. R News. 2008;8: 8–11.

[62] R Development Core Team R: A language and environment for statistical computing In: Computing RFfS, editor 3.0.2 ed 2011 Vienna, Austria.

[63] Pyšek P, Pyšek A. Invasion by Heracleum mantegazzianum in different habitats in the Czech Republic. J Veg Sci. 1995;6: 711–718.

[64] Usher MB, Kornberg H, Horwood JW, Southwood R, Moore PD. Invasibility and wildlife conservation: invasive species on nature reserves. Proc R Soc Lond B Biol Sci. 1986;314: 695–710.

[65] Bjerknes A-L, Totland O, Hegland SJ, Nielsen A. Do alien plant invasions really affect pollination success in native plant species? Biol Conserv. 2007;138: 1–12.

[66] Morales CL, Traveset A. A meta-analysis of impacts of alien vs. native plants on pollinator visitation and reproductive success of co-flowering native plants. Ecol Lett. 2009;12: 1–13. 10.1111/j.1461-0248.2009.01319.x 19453616

[67] Dietzsch AC. Impacts of the alien invasive Rhododendron ponticum L. on native plants, pollinators and their interaction. 2009. PhD Thesis, Trinity College Dublin.

[68] Goulson D. Are insects flower constant because they use search images to find flowers? Oikos. 2000;88: 547–552.

[69] Knight ME, Osborne JL, Sanderson RA, Hale RJ, Martin AP, et al Bumblebee nest density and the scale of available forage in arable landscapes. Insect Conserv Divers. 2009;2: 116–124.

[70] Albrecht M, Padrón B, Bartomeus I, Traveset A. Consequences of plant invasions on compartmentalization and species’ roles in plant—pollinator networks. Proc R Soc Lond B Biol Sci. 2014;281: 20140773. 10.1098/rspb.2014.0773 PMC4083793 24943368

[71] Burkle LA, Alarcón R. The future of plant—pollinator diversity: understanding interaction networks across time, space, and global change. Am J Bot. 2011;98: 528–538. 10.3732/ajb.1000391 21613144

[72] Alarcón R, Waser NM, Ollerton J. Year‐to‐year variation in the topology of a plant—pollinator interaction network. Oikos. 2008;117: 1796–1807.

[73] Dupont YL, Padrón B, Olesen JM, Petanidou T. Spatio‐temporal variation in the structure of pollination networks. Oikos. 2009;118: 1261–1269.

[74] Petanidou T, Kallimanis AS, Tzanopoulos J, Sgardelis SP, Pantis JD. Long‐term observation of a pollination network: fluctuation in species and interactions, relative invariance of network structure and implications for estimates of specialization. Ecol Lett. 2008;11: 564–575. 10.1111/j.1461-0248.2008.01170.x 18363716

[75] Clout MN, Veitch CR. Turning the tide of biological invasion: the potential for eradicating invasive species. Auckland, NZ IUCN Species Survival Commission 2002 Available: http://www.pacificinvasivesinitiative.org/site/pii/files/resources/publications/other/turning_the_tide.pdf. Accessed March 2014.

[76] Ferrero V, Castro S, Costa J, Acuña P, Navarro L, et al Effect of invader removal: pollinators stay but some native plants miss their new friend. Biol Invasions. 2013;15: 2347–2358.

