# Supplementary material for: Insect-Flower Interaction Network Structure Is Resilient to a Temporary Pulse of Floral Resources from Invasive Rhododendron ponticum
Source: PLoS One. 2015 Mar 12;10(3):e0119733. doi: 10.1371/journal.pone.0119733 (PMC4357452; doi:10.1371/journal.pone.0119733)
Supplement: S1 Table — Species codes and long hand for pollinator guilds represented in Fig. 5. (DOCX) [file pone.0119733.s001.docx]

**S1 Table**

| X1 | *Andrena* |
| --- | --- |
| X2 | *Apis mellifera* |
| X3 | *Baccha elongata* |
| X4 | *Bombus hortorum* |
| X5 | *Bombus jonellus* |
| X6 | *Bombus lucorum aggregate* |
| X7 | *Bombus pascuorum* |
| X8 | *Bombus pratorum* |
| X9 | *Episyrphus balteatus* |
| X10 | *Eriozona syrphoides* |
| X11 | *Eristalis pertinax* |
| X12 | *Lasioglossum* |
| X13 | *Leucozona lucorum* |
| X14 | *Melanostoma/Platycheirus* |
| X15 | *Meliscaeva* |
| X16 | *Parasyrphus annulatus* |
| X17 | *Pieris napi* |
| X18 | *Rhingia campestris* |
| X19 | *Sericomyia lappona* |
| X20 | *Sericomyia silentis* |
| X21 | *Sphegina clunipes* |
| X22 | *Syrphus* |
| X23 | Unidentified moth |
| X24 | Unidentified syrphid |
| X25 | *Volucella pellucens* |
| X26 | *Xylota* |
