# Supplementary material for: Insect-Flower Interaction Network Structure Is Resilient to a Temporary Pulse of Floral Resources from Invasive Rhododendron ponticum
Source: PLoS One. 2015 Mar 12;10(3):e0119733. doi: 10.1371/journal.pone.0119733 (PMC4357452; doi:10.1371/journal.pone.0119733)
Supplement: S2 Table — Species codes and long hand for plants represented in Fig. 5. (DOCX) [file pone.0119733.s002.docx]

**S2 Table.**

| 1 | *Rhododendron ponticum* |
| --- | --- |
| 2 | *Leycesteria formosa* |
| 3 | *Cardamine pratensis* |
| 4 | *Cirsium palustre* |
| 5 | *Conopodium majus* |
| 6 | *Cytisus scoparius ssp. scoparius* |
| 7 | *Digitalis purpurea* |
| 8 | *Filipendula ulmaria* |
| 9 | *Galium aparine* |
| 10 | *Geranium robertianum* |
| 11 | *Glechoma hederacea* |
| 12 | *Hyacinthoides non-scripta* |
| 13 | *Hypericum androsaemum* |
| 14 | *Hypericum pulchrum* |
| 15 | *Mimulus guttatus* |
| 16 | *Lonicera periclymenum* |
| 17 | *Lysimachia nemorum* |
| 18 | *Meconopsis cambrica* |
| 19 | *Potentilla reptans* |
| 20 | *Ranunculus repens* |
| 21 | *Rubus fruticosus* |
| 22 | *Sambucus nigra* |
| 23 | *Scrophularia nodosa* |
| 24 | *Sorbus aucuparia* |
| 25 | *Stachys sylvatica* |
| 26 | *Stellaria graminea* |
| 27 | *Stellaria holostea* |
| 28 | *Stellaria palustris* |
| 29 | *Taraxacum agg.* |
| 30 | *Trifolium pratense* |
| 31 | *Trifolium repens* |
| 32 | *Ulex europaeus* |
| 33 | *Valeriana dioica* |
| 34 | *Veronica chamaedrys* |
| 35 | *Vicia sepium* |
