# Supplementary material for: Insect-Flower Interaction Network Structure Is Resilient to a Temporary Pulse of Floral Resources from Invasive Rhododendron ponticum
Source: PLoS One. 2015 Mar 12;10(3):e0119733. doi: 10.1371/journal.pone.0119733 (PMC4357452; doi:10.1371/journal.pone.0119733)
Supplement: S3 Table — The contribution of each plant species to the composition of floral resources at sites invaded by R. ponticum in round 1 vs. 2 of sampling as determined by SIMPER (Similarity of Percentages) analysis. Data were square root transformed. (DOCX) [file pone.0119733.s003.docx]

**S3 Table.**

| Species | Average Abundance R1 | Average Abundance R2 | Percent contribution | Cumulative percent |
| --- | --- | --- | --- | --- |
| *Rhododendron ponticum* | 22.48 | 0 | 22.17 | 22.17 |
| *Stachys sylvatica* | 0 | 6.3 | 6.61 | 28.78 |
| *Hyacinthoides non-scripta* | 6.78 | 0 | 6.41 | 35.19 |
| *Digitalis purpurea* | 1.03 | 6.54 | 5.81 | 41 |
| *Rubus fruticosus* | 0 | 5.79 | 5.77 | 46.77 |
| *Veronica chamaedrys* | 4.64 | 1.01 | 4.19 | 50.96 |
| *Galium aparine* | 0 | 4.13 | 4.05 | 55.01 |
| *Stellaria graminea* | 0 | 3.17 | 3.37 | 58.38 |
| *Hypericum androsaemum* | 0.2 | 3.22 | 2.97 | 61.35 |
| *Lonicera periclymenum* | 0.22 | 3.01 | 2.94 | 64.29 |
| *Trifolium repens* | 0 | 2.7 | 2.74 | 67.04 |
| *Ranunculus repens* | 3.78 | 3.54 | 2.7 | 69.73 |
| *Scrophularia nodosa* | 0.37 | 2.84 | 2.63 | 72.37 |
