# Supplementary material for: Insect-Flower Interaction Network Structure Is Resilient to a Temporary Pulse of Floral Resources from Invasive Rhododendron ponticum
Source: PLoS One. 2015 Mar 12;10(3):e0119733. doi: 10.1371/journal.pone.0119733 (PMC4357452; doi:10.1371/journal.pone.0119733)
Supplement: S4 Table — The contribution of each insect taxon to communities at sites invaded by R. ponticum in round 1 vs. 2 of sampling, as determined by SIMPER (Similarity of Percentages) analysis. Data were square root transformed. (DOCX) [file pone.0119733.s004.docx]

**S4 Table.**

| Species | Mean interaction frequency R1 | Mean interaction frequency R2 | Percent contribution | Cumulative percent |
| --- | --- | --- | --- | --- |
| *Meliscaeva* | 0.46 | 0.95 | 12.95 | 12.95 |
| *Bombus lucorum aggregate* | 1.01 | 0.36 | 10.91 | 23.86 |
| *Sphegina clunipes* | 1.08 | 0.41 | 9.47 | 33.33 |
| *Bombus pratorum* | 0.65 | 0.11 | 8.66 | 41.99 |
| *Eristalis pertinax* | 0.37 | 0.44 | 6.52 | 48.51 |
| *Episyrphus balteatus* | 0.31 | 0.35 | 4.91 | 53.42 |
| *Sericomyia silentis* | 0.19 | 0.29 | 4.88 | 58.3 |
| *Syrphus* | 0.33 | 0.57 | 4.66 | 62.96 |
| *Eriozona syrphoides* | 0.36 | 0 | 4.57 | 67.53 |
| *Xylota* | 0.32 | 0.13 | 3.59 | 71.13 |
